# Combined Salvianolic Acid B and Ginsenoside Rg1 Exerts Cardioprotection against Ischemia/Reperfusion Injury in Rats

**DOI:** 10.1371/journal.pone.0135435

**Published:** 2015-08-17

**Authors:** Yanping Deng, Min Yang, Feng Xu, Qian Zhang, Qun Zhao, Haitao Yu, Defang Li, Ge Zhang, Aiping Lu, Kenka Cho, Fukang Teng, Peng Wu, Linlin Wang, Wanying Wu, Xuan Liu, De-an Guo, Baohong Jiang

**Affiliations:** 1 State Key Laboratory of Drug Research, Shanghai Institute of Materia Medica, Chinese Academy of Sciences, Haike Road #501, Shanghai, 201203, China; 2 Shenyang Pharmaceutical University, Wenhua Road #103, Shenyang, 110016, China; 3 Department of Medicinal Chemistry School of Pharmacy, Fudan University, Shanghai, 200237, China; 4 Institute for Advancing Translational Medicine in Bone and Joint Diseases, School of Chinese Medicine, Hong Kong Baptist University, Hong Kong SAR, China; 5 Takarazuka University of Medical and Health Care, Hanayashiki-Midorigaoka, Takarazuka-city, 6660162, Japan; Emory University, UNITED STATES

## Abstract

Lack of pharmacological strategies in clinics restricts the patient prognosis with myocardial ischemia/reperfusion (I/R) injury. The aim of this study was to evaluate the cardioprotection of combined salvianolic acid B (SalB) and ginsenoside Rg1 (Rg1) against myocardial I/R injury and further investigate the underlying mechanism. I/R injury was induced by coronary artery ligation for Wistar male rats and hypoxia/reoxygenation injury was induced on H9c2 cells. Firstly, the best ratio between SalB and Rg1was set as 2:5 based on their effects on heart function detected by hemodynamic measurement. Then SalB-Rg1 (2:5) was found to maintain mitochondrial membrane potential and resist apoptosis and necrosis in H9c2 cell with hypoxia/reoxygenation injury. Companying with same dose of SalB or Rg1 only, SalB-Rg1 showed more significant effects on down-regulation of myocardial infarct size, maintenance of myocardium structure, improvement on cardiac function, decrease of cytokine secretion including TNF-α, IL-1β, RANTES and sVCAM-1. Finally, the SalB-Rg1 improved the viability of cardiac myocytes other than cardiac fibroblasts in rats with I/R injury using flow cytometry. Our results revealed that SalB-Rg1 was a promising strategy to prevent myocardial I/R injury.

## Introduction

Myocardial infarction is the major cause of death and disability worldwide [1, 2]. Timely restoration of coronary bloodstream via percutaneous coronary intervention (PCI) is currently the guideline-recommended treatment for patients with acute myocardial infarction [3,4]. However, ischemia/reperfusion (I/R) injury, which is known as cardiac ischemia followed by PCI, has been shown to often result in contractile dysfunction because of increased cell death and infarction size [5, 6]. Thus, novel therapeutic medicine is urgent needed to limit the extent of I/R injury after myocardial infarction.

Currently, clinical treatments always focus on immediate recanalization despite the known subsequent damage following reperfusion, addressing therapeutic significance to target I/R injury. Different mechanisms including oxidative stress, inflammatory response, apoptosis and metabolism contribute to this injury [7]. Thus targeting any single molecule in above-mentioned mechanisms individually will have limited clinical impact. A strategy focusing on cellular function as a whole rather than each individual molecule within the pathway may increase therapeutic potency.

The herb pair, derived from roots of *Salviae miltiorrhizae* (Danshen in Chinese) and *Panax notoginseng* (Sanqi in Chinese), has been widely used for improving coronary or cerebral circulation in China as well as in western countries [8]. Many kinds of commercially available preparations containing this herb pair, known as Danshen formulae, have been marketed for a long time and ranked as the first-line drugs among all Traditional Chinese Medicines (TCM) in China [9,10]. Therefore, it is a very essential question to explore why and how the combination of *Salviae miltiorrhizae* and *Panax notoginseng* considerably outperforms individual herb on cardioprotection.

In the present study, we conducted an experimental I/R injury model to evaluate the cardioprotection for combination of salvianolic acid B (SalB), the main active ingredient of *Salviae miltiorrhizae*, with ginsenoside Rg1 (Rg1), the main active ingredient of *Panax notoginseng*. Furthermore, a series of assays were employed to explore the potential cardioprotective mechanism of this combination through biochemical, functional and histopathological analysis.

## Materials and Methods

### Reagents

SalB and Rg1 were purchased from Shanghai Yousi Bio-Tech Co., Ltd (Shanghai, China). The structure of SalB (S1 Fig) or Rg1 (S2 Fig) was elucidated by ^1^H and ^13^C NMR spectrum using Bruker AM-400 spectrometer. Purity of SalB was 99.84%, and Rg1 was 99.78% evaluated by high performance liquid chromatography (S3 Fig). Procarta cytokine profiling kit (Panomics, CA, USA), chloral hydrate (SCRC, Shanghai, China), 3,5-triphenyltetrazolium chloride (Sigma-Aldrich, USA), troponin-I antibody (abcam, Cambridge, United Kingdom), vimentin antibody (Calbiochem, Darmstadt, Germany), β-actin antibody (Cell-signaling, Boston, USA) and collagenase Type II (DingGuo, Shanghai, China) were commercially obtained. Mitochondrial membrane potential assay kit was purchased from Beyotime (Jiangsu, China). H9c2 cells were purchased from Cell bank of Chinese Academy of Sciences (Shanghai, China). All the culture reagents were products from Thermo Scientific (Beijing, China) unless specified otherwise.

### Rat model of ischemia/reperfusion injury

This study was approved by the Animal Care and Use Committee at Shanghai Institute of Materia Medica (IACUC number: SIMM-AE-GDA-2010-06) and followed with the Guide for the Care and Use of Laboratory Animals published by the National Institutes of Health. Wistar male rats (250~300 g) were kept in a temperature-controlled room (22 ± 2°C) with 12 hours light and dark cycle. Water and diet were available ad libitum. Briefly, after anesthesia with chloral hydrate (330 mg/kg) through intraperitoneal injection and connection to a ALC-9 ventilator (Shanghai ALCBio,China), rat heart was exposed via a left thoracotomy at the fourth intercostal space followed by a ligature below the left descending coronary artery. Regional left ventricular ischemia was confirmed by bleaching of the myocardium and elevation of ST-segment in the electrocardiogram. The ventricular ischemia was maintained for 40 min following with reperfusion that was initiated by releasing the ligature for 60 min (S4 Fig). Sham-operated mice underwent a similar procedure without ligation. During the process of ischemia/reperfusion, animals were monitored carefully and 50 mg/kg ketamine was injected intraperitoneally before hemodynamic detection to minimize the pain caused by the surgery.

To detect the best ratio between SalB and Rg1, rats were divided into the following groups: Sham-operated rats were given saline (Sham); ischemia-reperfusion rats were given saline (I/R); ischemia-reperfusion rats were treated with 10 mg/kg SalB-Rg1 combination, the weight ratio for SalB to Rg1 was 1:5, 2:5, 3:5, 4:5 and 5:5, respectively. SalB-Rg1 combination was administered at the same time of reperfusion. More than 6 rats were used for each group.

After that the optimal ratio for SalB to Rg1 was set as 2:5, we evaluated the cardioprotection and elucidated the underlying mechanism of SalB-Rg1 combination, and further compared the effects of SalB-Rg1 combination (2:5) with single SalB or single Rg1 at the same dose. Animals were randomly assigned into five groups: Sham-operated rats were given saline (Sham); ischemia-reperfusion rats were given saline (I/R); ischemia-reperfusion rats were given 10 mg/kg SalB (I/R-SalB); ischemia-reperfusion rats were given 10 mg/kg Rg1 (I/R-Rg1); ischemia-reperfusion rats were given 10 mg/kg SalB-Rg1 [I/R-(SalB-Rg1)]. The exact dose of SalB was 2.8 mg/kg and Rg1 was 7.2 mg/kg in 10 mg/kg SalB-Rg1 combination. The number of animals in each group was more than 25 to keep the animal number more than 6 for each test item such as hemodynamic measurement, histopathological detection and flow cytometry assay.

### Hemodynamic measurement

Hemodynamic measurement was followed closely with ischemia/reperfusion, 330 mg/kg chloral hydrate and 50 mg/kg ketaimine could maintain anesthesia for rats during the whole procedure. Briefly, a polyethylene tube (OD 0.90 mm X ID 0.50 mm) was inserted into left ventricle through right carotid artery. Heart rate, mean arterial pressure (MAP), left ventricular systolic pressure (LVSP) and end-diastolic pressure (EDP) were recorded by PowerLab 8/30 instrument (ADInstruments, Australia). Maximal rate of pressure development for contraction (+dP/dt_max_) and maximal rate of pressure development for relaxation (–dP/dt_max_) were all calculated from the continuously collected pressure signal as we previous report [11].

### Hypoxia/reoxygenation injury on H9c2 cells

To detect the direct protection of SalB-Rg1 combination at the ratio of 2:5 on H9c2 cell, H9c2 cells were cultured in DMEM supplemented with 10% FBS and maintained in 5% CO_2_ at 37°C. For induction of hypoxia/reoxygenation (H/R) injury, H9c2 cells were incubated with fresh medium without FBS and put into a three-gas incubator (Heal Force, Shanghai, China) to achieve hypoxia environment (95% N_2_ and 5% CO_2_) for 24 hours. Then, re-oxygenation was initiated by changing culture medium with 10% FBS and moving to an incubator (5% CO_2_,) at 37°C. SalB, Rg1 or SalB-Rg1 combination was treated at the time of re-oxygenation, and the final concentration of different components was 0.1 μM. Control cell was cultured with 10% FBS medium in an incubator (5% CO_2_) at 37°C during the whole experiment. All experiments using H9c2 cells were repeated at least three times.

### Measurement of mitochondrial membrane potential *in vitro*

The loss of the mitochondrial membrane potential which occurs during apoptosis was evaluated by a commercial kit with 5,5’,6,6’-tetrachloro-1,1’,3,3’- tetraethyl-imidacarbocyanine iodide (JC-1). In normal cells, JC-1 forms multimers fluorescencing red. In apoptotic cells, JC-1 forms monomers with green florescence. In the present study, H9c2 cells were incubated with 2 mg/ml JC-1 for 10 min at 37°C after 24 h hypoxia and 24 h re-oxygenation. Green fluorescence of JC-1 monomer was observed by examination at 488 nm excitation and 530 nm emission. Red fluorescence of JC-1 multimer was observed at the emission at 590 nm. Photomicrographs were taken using a BX51 microscope with DP71 CCD camera (Olympus Corporation). More than twelve areas in three separated cultured dishes were scanned and the average intensity for each region was quantified. The ratio of JC-1 red to green fluorescence intensity for each region was calculated. A decrease in this ratio was interpreted as decrease of mitochondrial membrane potential, whereas an increase in the ratio was interpreted as gain in mitochondrial membrane potential.

### Cells death detection *in vitro*

The death of H9c2 cells was determined with Annexin V-FITC/PI Apoptosis Detection Kit according manufacturer’s protocol after 24 h hypoxia and 46 h re-oxygenation. In brief, H9c2 cells were washed twice with PBS, and then immersed in 100 μl binding buffer, followed by staining with 5 μl Annexin V-FITC and 10 μl propidium iodide (PI) for 15 min at room temperature in the dark. The status of cell fluorescence was observed by Olympus BX51 microscope and photomicrographs were taken with Olympus DP71 CCD camera.

### Determination of myocardial infarct size

Animals were anesthetized with 330 mg/kg chloral hydrate and 50 mg/kg ketaimine intraperitoneally, rat hearts were isolated after hemodynamics measurements. Myocardial infarct size was detected using 2,3,5-Triphenyltetrazolium chloride (TTC) staining after hemodynamics measurements. The hearts were washed with cold saline and cut into 2-mm slices. Sections were incubated in 5 ml of 0.1% TTC for 20 minutes at 37°C. After fixation in 10% formaldehyde for 24 hours, the slices were photographed by Olympus BX51 microscope. The red regions represent non-infarct tissue, and pale white regions display infarct tissue. Morphometric measurement of infarct area performed by image analysis system (Image-Pro Plus version 6.0.).

### Histopathological detection

Hearts from each group were fixed with neutral formalin (10%) for 48 hours and embedded in paraffin. Then the sample were cut into 3 μm-thick sections and stained with hematoxylin and eosin. Photomicrographs were taken using a BX51 microscope with DP71 CCD camera (Olympus Corporation).

### Multiplex cytokine assay

Procarta cytokine profiling kit was used to detect 6 different rat cytokines per reaction for protein extracted from myocardial infarct area. This assay uses xMAP technology to enable the detection and quantification of multiple protein targets simultaneously [12]. Firstly, multiple samples from myocardial infarct area were homogenized simultaneously using a FastPrep tissue homogenizer (MP Biomedicals, CA, USA) for cytokines assay. Then, 50 μl/well antibody beads were added onto the Filter plate, washed by Wash buffer. Then, 50 μl/well of each sample was added, incubated for at least 1 h at room temperature and washed with Wash buffer. Afterwards, 25 μl/well of the Detection Antibody was added and the Filter plate was shaken at 500 rpm for 30 min at room temperature. After adding Sreptavidin-PE, the detection was conducted using a Luminex 200 instrument (Bio-Rad, CA, USA). In the present study, investigated cytokines were tumor necrosis factor-α (TNF-α), interleukin-1β (IL-1β), intercellular adhesion monocyte chemoattractant protein-3 (MCP-3), regulated on activation normal T cell expressed and secreted (RANTES), intercellular adhesion molecule (ICAM), soluble vascular cell adhesion molecule-1 (sVCAM-1).

### Heart perfusion and enzyme digestion

Adult rat heart cells were isolated essentially as described previously with little modification. Rats were anesthetized with 350 mg/kg chloral hydrate intraperitoneally and heparinized (1000 IU/kg) intravenously. Hearts were rapidly excised and perfused on a Langendorff apparatus with cold Hank’s solution containing (in mM) 137 NaCl, 0.44 KH_2_PO_4_, 4.16 NaHCO_3_, 5.37 KCl, 0.33 NaH_2_P0_4_, 5.5 glucose, 1.26 CaCl_2_, 0.73 MgSO_4_ with pH 7.4 at 37°C, following 5 min with a D-Hank’s solution (without CaCl_2_ and MgSO_4_). Then, collagenase Type II (0.5 mg/ ml) and trypsin (0.1 mg /ml) was added for additional 8 min. All the liberated cells were collected and re-suspended in DMEM containing 10% FBS at 37°C for 40 min.

### Identification of cardiac myocytes and fibroblasts by western blot analyses

Cardiac fibroblasts attached to the bottom of the culture dish during 40 min incubation were collected firstly. Whereas non-adherent cells were removed to a new culture dish for further 50 min, and non-adherent cardiac myocytes were collected then. Suspension cells or adherent cells were identified by western blot analyses. Vimentin is the marker protein for cardiac fibroblasts and troponin-I is the marker protein for cardiac myocytes. Briefly, the aliquots of 20 μg protein were subjected to SDS-PAGE and were transferred onto polyvinylidene difluoride (PVDF) membranes. The membranes were blocked for 2 h with 5% skimmed milk in Tris-buffered saline with 0.1% Tween-20 (pH 7.4) at 37°C, then incubated with primary antibodies against Troponin-I (1:100),vimentin (1:100) or β-actin overnight at 4°C. After that, the membrane was incubated with HRP-conjugated second antibody at room temperature for 2 h. The immunoreactive bands were detected by chemiluminescence methods and visualized on Kodak Omat X-ray films. Densitometric analysis of the images obtained from X-ray films was performed with MiniBis pro (DNR Bio-Imaging Systems Ltd. Jerusalem, Israel).

### Establishment of the gating strategy for cardiac myocytes and fibroblasts by flow cytometry

After suspension cells were identification as cardiac myocytes and adherent cells were identified as cardiac fibroblasts by western blot. The gating strategy for cardiac myocytes or cardiac fibroblasts without labeling was further established by the forward (FSC) and side scatter (SSC) through flow cytometry (BD Biosciences, MD, USA). The gating position of cardiac myocytes or cardiac fibroblasts was defined in SSC and FSC according their different cell size separately.

### Cardiac protection detected by flow cytometry

After confirming the gating position of cardiac myocytes and cardiac fibroblasts based on the FSC and SSC using flow cytometry, the effects of SalB-Rg1 combination on viability of cardiac myocytes or cardiac fibroblasts was evaluated on rat with ischemia/reperfusion injury subsequently. After surgery, rat hearts from different treatment were enzymolysis using Langendorff apparatus. The total cardiac cells was labeled using Annexin V-FITC/PI Apoptosis Detection Kit and the viability was evaluated through flow cytometry. Briefly, isolated cells from Langendorff perfusion, which included cardiac myocytes and cardiac fibroblasts, were adjusted to 1×10^6^ cells/mL. Then cells were immersed in 100 μl binding buffer, and stained with 5 μl of Annexin V-FITC for 15 min at room temperature in the dark. Subsequently, the cells were stained with 10 μl PI and detected by flow cytometry. At last, cell viability was calculated as the percentage of (PI/Annexin negative cells) to total cells using WinMDI 2.9 software.

### Statistical analysis

All quantitative values were expressed as mean ± S.E and analyzed by SPSS 18.0 software (SPSS Inc., Chicago, IL, USA). Mean values of data from different groups were compared using one-way ANOVA. After confirming the equal variances, least-significant difference (LSD) was used to compare the difference between two groups. *P* < 0.05 was considered to be statistically significant.

## Results

### The ratio for SalB to Rg1 was set as 2:5 based on left ventricular function

**Fig 1:**
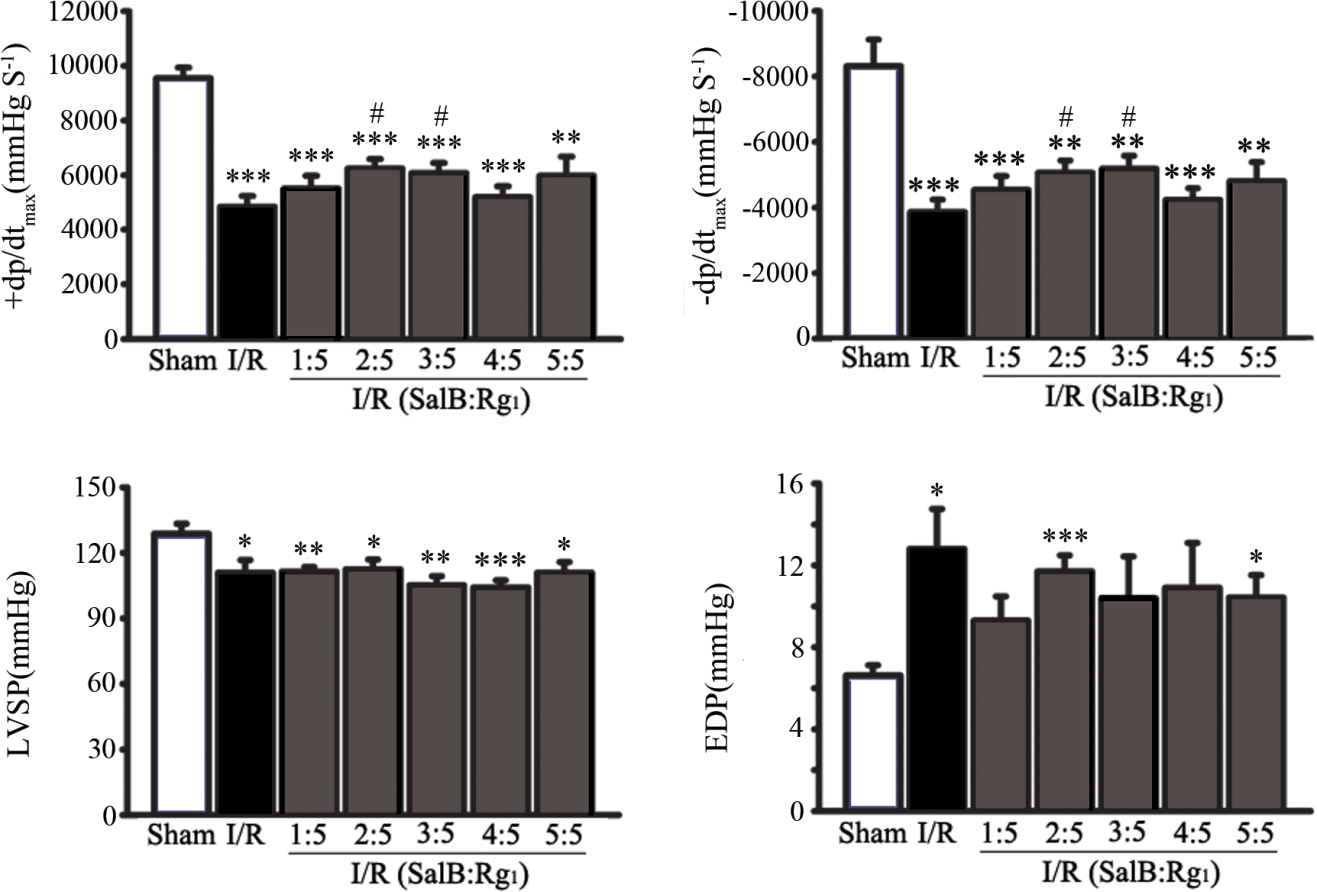
The ratio for SalB to Rg1 was set as 2:5 based on ventricular function. I/R injury was induced by 40 minutes coronary artery ligation followed by 1 hour reperfusion. Ventricular function was evaluated by hemodynamic parameters including maximum rate of pressure development for contraction (+dP/dt_max_), maximum rate of pressure development for relaxation (-dP/dt_min_), left ventricular systolic pressure (LVSP) and end-diastolic pressure (EDP). All the values were expressed as mean ± S.E. *p<0.05, **p<0.01, ***p<0.001 versus Sham; #p<0.05 versus I/R; n≥6 for every group.

Rat model for ischemia/reperfusion injury was used to detect the optimal ratio for SalB to Rg1. The protective effect of SalB-Rg1 combination with different ratio was evaluated by several major haemodynamic parameters for left ventricle function, including +dp/dt_max_,-dp/dt_max_, LVSP and EDP (Fig 1). Compared to rats in the Sham group, left ventricle dysfunction in I/R rats was observed with significant decrease of +dp/dt_max_ (4839.1 ± 1039.0 mmHgS^-1^ versus 9549.8 ± 941.7 mmHgS^-1^, p<0.001),-dp/dt_max_ (-3864.8 ± 983.2 mmHgS^-1^ versus -8314.2 ± -1981.3 mmHgS^-1^, p<0.001) and LVSP (110.8 ± 15.6 mm Hg versus 128.8 ± 11.2 mm Hg, p<0.05); increase of EDP (12.8 ± 5.1 mmHg versus 6.6 ± 1.2 mmHg, p<0.05). SalB-Rg1 treatment at the ratio of 2:5 reduced the degree of impairment of left ventricle function with the elevation of values of +dp/dt_max_ (6237.9 ± 986.9 mmHgS^-1^ versus 4839.1 ± 1039.0 mmHgS^-1^, p<0.05) and-dp/dt_max_ (-5062.8 ± 1038.2 mmHgS^-1^ versus -3864.8 ± 983.2 mmHgS^-1^, p<0.05) compared with the I/R group. SalB-Rg1 treatment at the ratio of 3:5 reduced the degree of impairment of left ventricle function with the elevation of values of +dp/dt_max_ (6075.4 ± 901.2 mmHgS^-1^ versus 4839.1 ± 1039.0 mmHgS^-1^, p<0.05) and-dp/dt_max_ (-5178.7 ± 975.7 mmHgS^-1^ versus -3864.8 ± 983.2 mmHgS^-1^, p<0.05) compared with the I/R group. To further evaluate cardioprotection and underlying mechanism for combination of SalB and Rg1, the ratio of 2:5 was selected for the following experiment.

### SalB-Rg1 protected H9c2 cells against apoptosis and necrosis

**Fig 2:**
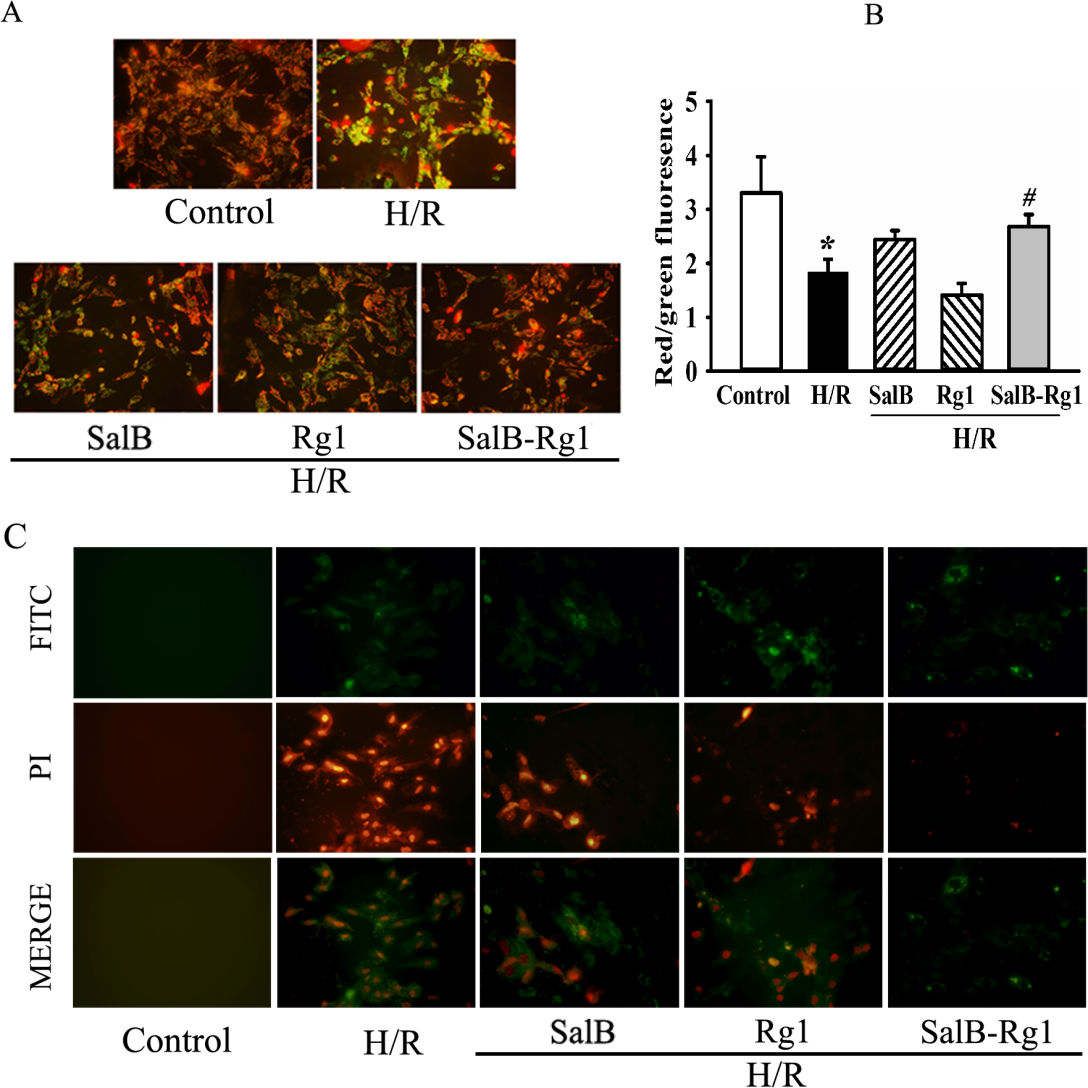
SalB-Rg1 protected H9c2 cells against apoptosis and necrosis. (A) The protection of SalB-Rg1 against H9c2 apoptosis was detected by mitochondrial depolarization using JC-1 stain. (B) Quantification of the ratio for JC-1 multimer to JC-1 monomer fluorescence intensity (red:green). (C) The apoptosis or necrosis of H9c2 cell was evaluated using Annexin V-FITC/PI Apoptosis Detection Kit. Apoptotic cells were stained green (Annexin V-FITC) and necrosis cells were stained red (PI). At least three time experiments were repeated and the representative figures were shown.

Cardiomyocyte apoptosis and necrosis play important roles in the pathology of ischemic/reperfusion injury. Mitochondrial membrane potential is an important index on cell apoptosis. The protection of SalB-Rg1 against apoptosis was detected through mitochondrial depolarization firstly (Fig 2A). In general, JC-1 forms multimers with red fluorescence in the normal cells which possessed normal mitochondrial membrane potential. In apoptotic cells, the mitochondrial membrane potential is depolarized and JC-1 forms monomers with green florescence. As shown, hypoxia/reoxygenation significantly enhanced green fluorescence in H9c2 cells. While cells treated by either SalB or Rg1 presented slight green fluorescence, and almost no green fluorescence was found in SalB-Rg1 treated cells. The ratios of JC-1 red to green fluorescence intensity for different treatments were quantified (Fig 2B). A decrease in this ratio was detected in H/R group, while this decrease was reversed in SalB-Rg1 group, suggesting considerate protection of this combination against apoptosis.

Then, apoptosis/necrosis was evaluated using Annexin V-FITC/PI Apoptosis Detection Kit (Fig 2C). Positive apoptotic cells were stained green (Annexin V-FITC) and necrosis cells were stained red (PI). The cell number was significant higher for both apoptosis and necrosis cells in H/R in comparison with control. SalB-Rg1 treatment not only down-regulated apoptosis but also necrosis cell number compared with H/R significantly.

### SalB-Rg1 improved cardiac function

**Fig 3:**
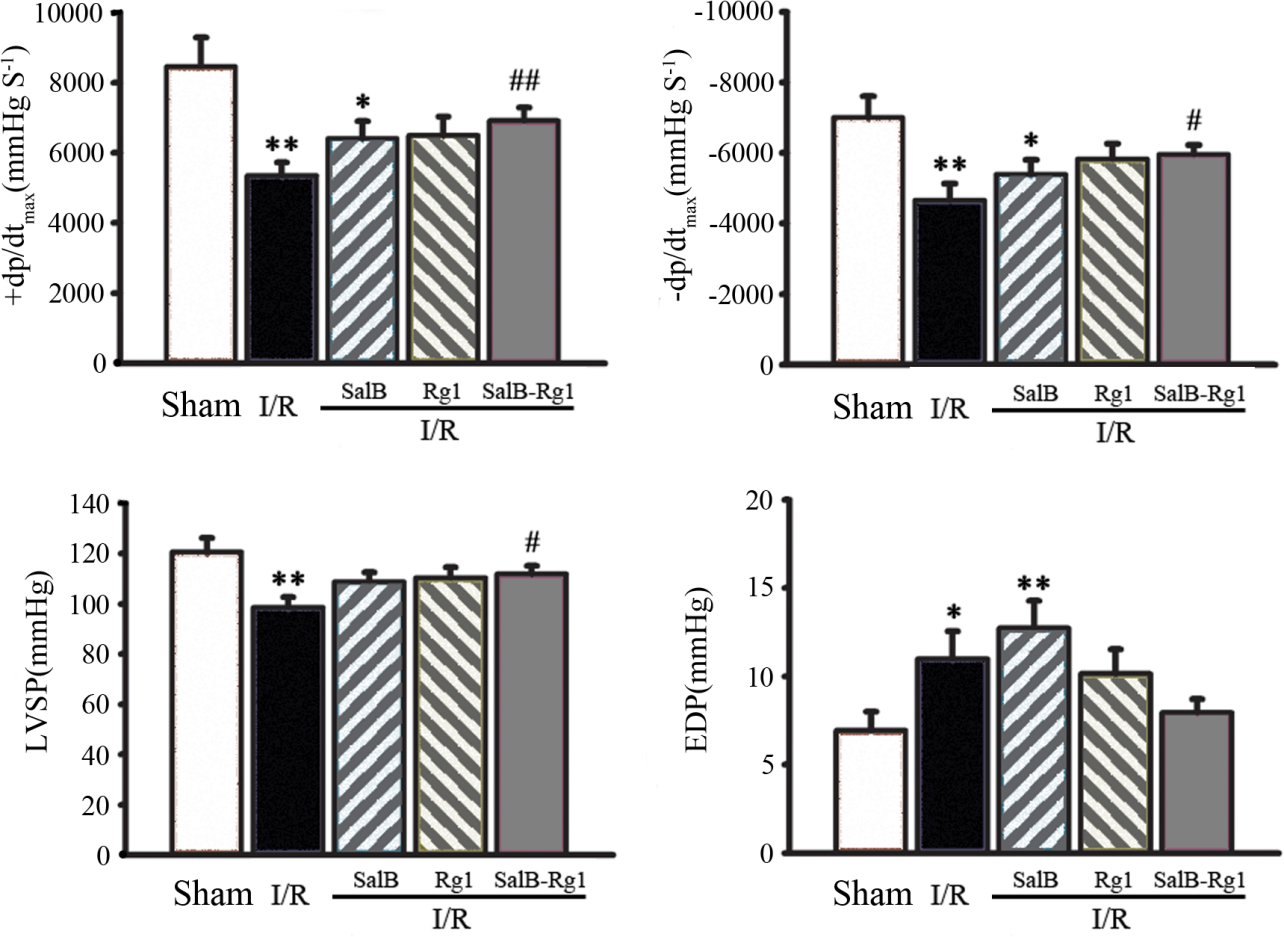
SalB-Rg1 (2:5) improved cardiac function. 10mg/kg SalB-Rg1 significantly improved ventricular function by evaluation of hemodynamic parameters including +dP/dt_max_,-dP/dt_min_, EDP and LVSP. No improvement on cardiac function was found with the treatment of SalB or Rg1 at 10 mg/kg only. All the values are expressed as mean ± S.E. *p<0.05, **p<0.01versus Sham; #p<0.05, ##p<0.01 versus I/R; n≥6 for every group.

To compare the cardio-protection of SalB-Rg1 (2:5) with single SalB or single Rg1 at the same dose, hemodynamic assay was conducted again (Fig 3). As compared with Sham group, left ventricle dysfunction in I/R rats was observed with significant decrease of +dp/dt_max_ (5347.1 ± 380.7 mmHgS^-1^ versus 8469.4 ± 821.4 mmHgS^-1^, p<0.01),-dp/dt_max_ (-4653.3 ± 471.9 mmHgS^-1^ versus -7013.71 ± -590.7 mmHgS^-1^, p<0.01) and LVSP (98.5 ± 4.2 mm Hg versus 120.5 ± 5.7 mm Hg, p<0.05); increase of EDP (11.0 ± 1.6 mmHg versus 6.9 ± 1.1 mmHg, p<0.05). SalB-Rg1 significantly improved cardiac function by increasing of +dP/dt_max_ (6917.5 ± 376.6 mmHgS^-1^ versus 5374.1 ± 380.7 mmHgS^-1^ P<0.01),-dP/dt_min_ (-5964.7 ± 253.2 mmHgS^-1^ versus -4653.3 ±471.9 mmHgS-^1^, P<0.05) and LVSP (111.8 ±3.3 mmHg versus 98.5 ± 4.2 mmHg, P<0.05) in comparison with I/R group. While no improvement on cardiac function was found with the treatment of single SalB or single Rg1 at the dose of 10 mg/kg.

### SalB-Rg1 down-regulated infarct area and protected cardiac structure

**Fig 4:**
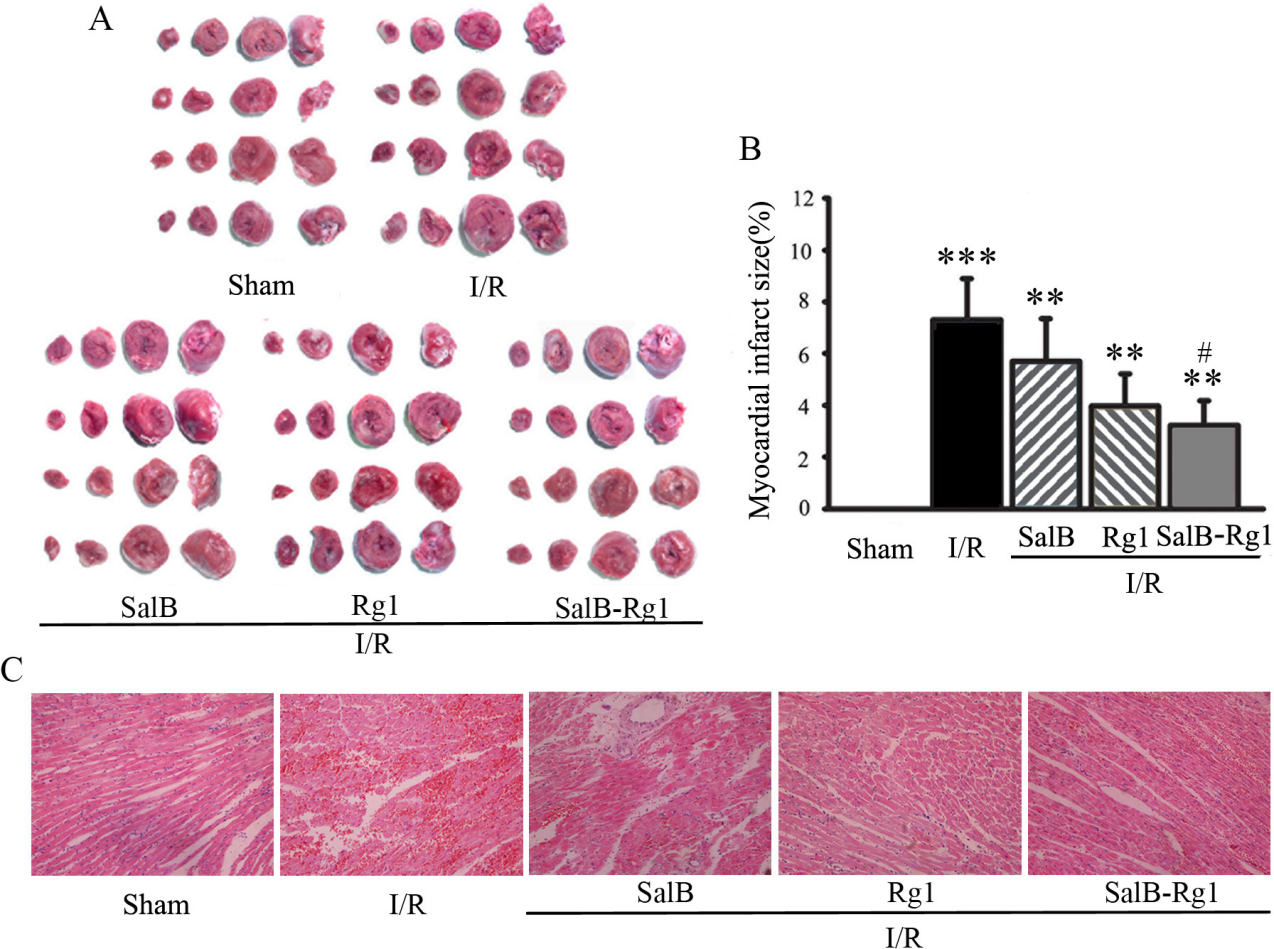
SalB-Rg1 down-regulated infarct area and protected cardiac structure. (A) Representative illustrations of heart sections stained with TTC. Infarct region was stained white. (B) The infarct size was evaluated as the percentage of infarct area to whole area. (C) Representative photomicrographs from heart tissue detected by H&E stain. All the values are expressed as mean ± S.E. **p<0.01, ***p<0.001 versus Sham; #p<0.05 versus I/R; n≥6 for every group.

I/R injury in rats were induced by 40 minutes ligation of the left descending coronary artery followed by 1 hour reperfusion. Histological analysis of infarct size was performed by TTC staining (Fig 4A). Fig 4B shows that the infarct area as a percentage of whole heart was significantly increased in the I/R group (7.3% ± 1.6%, p<0.001) in comparison with Sham. And SalB-Rg1 significantly decreased infarct area compared with the I/R group (3.2% ± 1.0% versus 7.3% ± 1.6%, p < 0.001). While, even downward trend of both SalB and Rg1 on infarct area was observed, no significant difference was found in comparison with I/R group.

To further explore the structural basis leading to observed impairment of left ventricle function, histopathological evaluation of heart samples from all groups was performed (Fig 4C). The cell alignment was regular and no obvious damage was detected in the myocardium of Sham rats. However, marked cellular degeneration, interstitial edema and coagulation necrosis in the ischemic region were observed in I/R rats. Upon SalB-Rg1 treatment, there was a profound increase of cross striation and cell integrity, suggesting that SalB-Rg1 markedly ameliorated the myocardial damage induced by I/R.

### SalB-Rg1 down-regulated inflammatory cytokines

**Fig 5:**
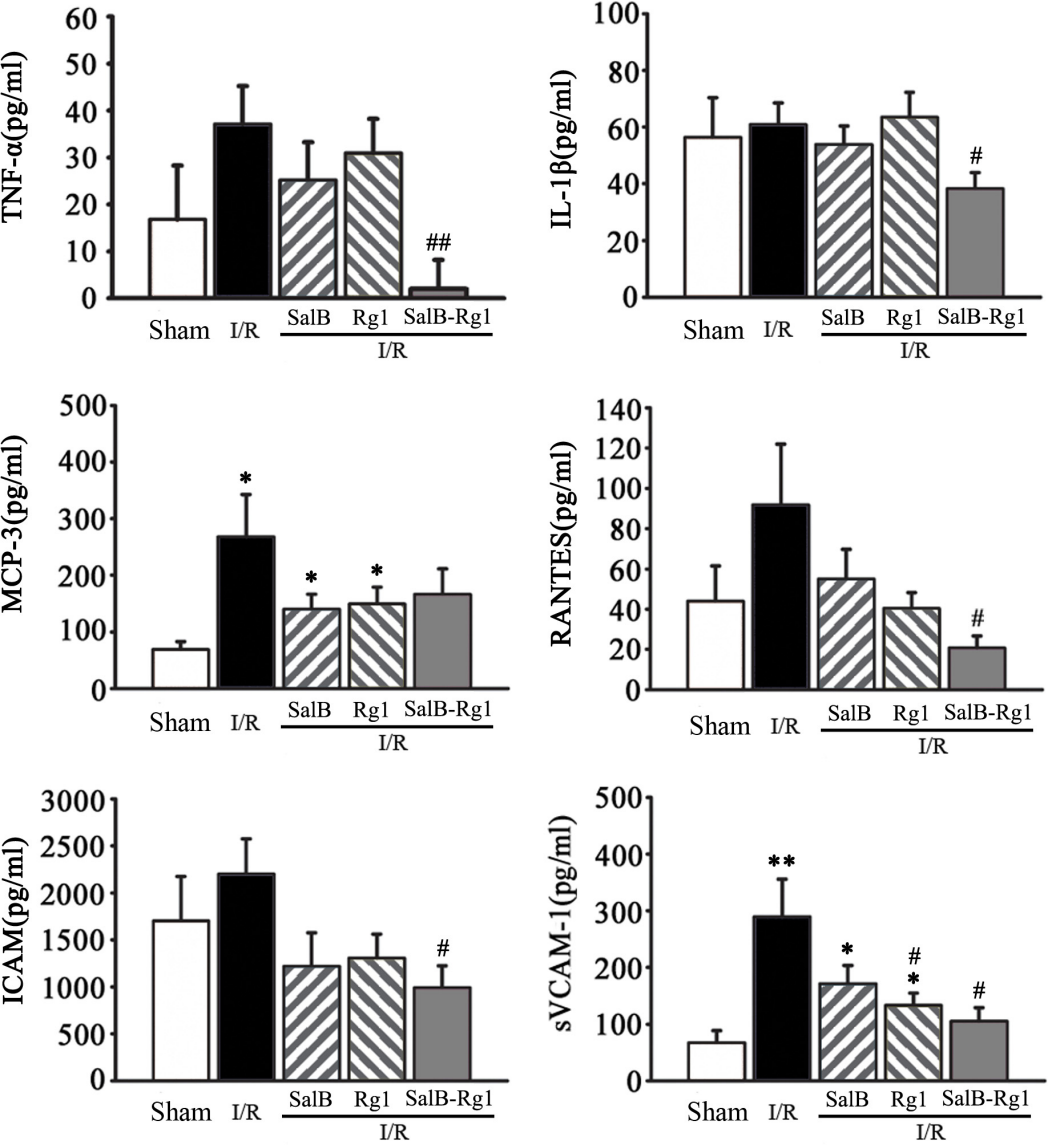
SalB-Rg1 down-regulated the secretion of cytokines. Six cytokines were detected by Procarta cytokine profiling assay. All the values are expressed as mean ± S.E. *p<0.05, **p<0.01 versus Sham; #p<0.05, ##p<0.01 versus I/R; n≥6 for every group.

To further characterize the influence of SalB-Rg1 on the secretion of inflammatory cytokines, 6 cytokines were evaluated in the Procarta cytokine profiling assay as shown in Fig 5. Remarkably, we observed that two cytokines, that are known to regulate chemotaxis and adhesion of inflammatory cells, were up-regulated with I/R injury. The two cytokines were MCP-3 (268.3± 74.1 versus 68.9 ± 14.0 pg/ml, p<0.05) and sVCAM-1 (289.6 ± 66.0 versus 67.3 ±21.4 pg/ml, p<0.05). SalB-Rg1 treatment down-regulated 4 cytokines in comparison with the I/R group, the 4 cytokines were TNF-± (2.0±6.1 pg/ml versus 37.1±8.13 pg/ml, p<0.05), IL-1β (38.4±5.6 pg/ml versus 60.9±7.6 pg/ml, p<0.05), RANTES (20.8±6.0 pg/ml versus 91.8±30.1 pg/ml, p<0.05) and sVCAM-1 (105.2±23.9 pg/ml versus 289.6±66.0, p <0.05). No regulation of SalB treatment on 6 cytokines was observed in comparison with I/R group. Rg1 only down regulated the expression level of sVCAM-1(p<0.05).

### SalB-Rg1 improved cell viability for cardiac myocytes instead of cardiac fibroblasts

**Fig 6:**
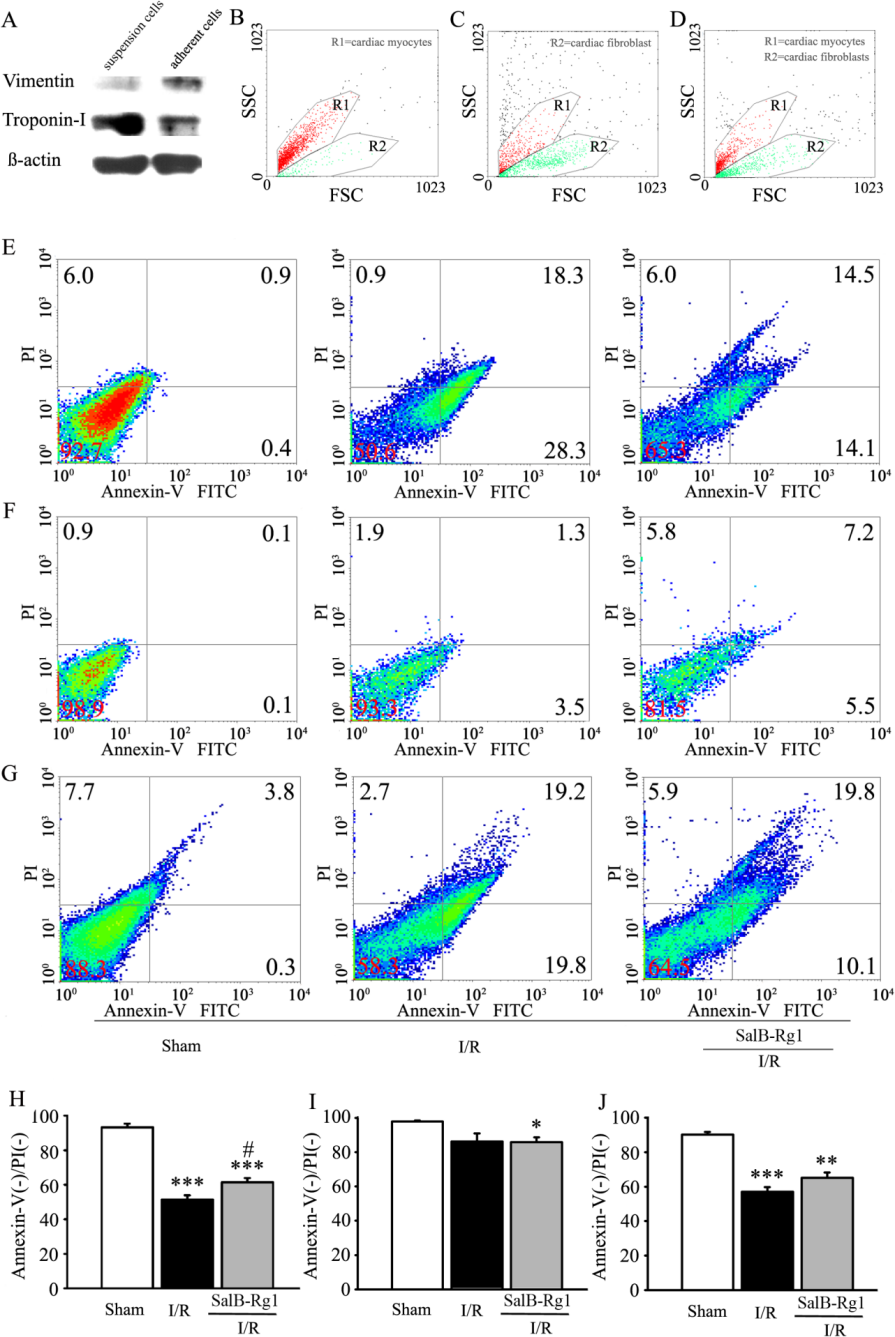
SalB-Rg1 improved cell viability for cardiac myocytes instead of cardiac fibroblasts. (A) Suspension cells (troponin-1I positive) were identified as cardiac myocytes and adherent cells (vimentin positive) were identified as cardiac fibroblasts by western blot. (B) Cardiac myocytes were positioned in gate R1 in scatter diagram by flow cytometry, (C) Cardiac fibroblasts were positioned in gate R2 in scatter diagram by flow cytometry, (D) Whole heart cells were positioned in gate R1 plus R2 in scatter diagram by flow cytometry. (E) Cardiac myocytes, (F) Cardiac fibroblast, (G) Total cells were analyzed by flow cytometry after stained with Annexin V-FITC/PI Apoptosis Detection Kit based on their gate position. (H) Cardiac myocytes, (I) Cardiac fibroblasts and (J) Whole heart cells were quantified for cell viability. All the values are expressed as mean ± S.E. *p<0.05, **p<0.01, ***p<0.001 versus Sham; #p<0.05 versus I/R; n≥6 for every group.

To further elucidate the cell type of SalB-Rg1 on cardioprotection, myocardial cells were isolated from whole heart using Langendoff apparatus. Cardiac myocytes and cardiac fibroblasts were further separated according their different velocity on adhesion. Suspension cells or adherent cells were identified by western blot (Fig 6A). Suspension cells showed troponin-I positive expression and were identified as cardiac myocytes, while adherent cells showed vimentin positive expression and were identified as cardiac fibroblasts.

To detected cell viability using flow cytometry, the position of cardiac myocytes (Fig 6B) or cardiac fibroblasts (Fig 6C) was defined in SSC and FSC, respectively. After that, the whole heart cells, including cardiac myocytes and cardiac fibroblasts (R1 plus R2), were gating again (Fig 6D). Gate R1 represented cardiac myocytes (suspension cells), and gate R2 represented cardiac fibroblasts (adherent cells).

Cell viability was conducted by flow cytometry using Annexin V-FITC/Propidium iodide (PI) Apoptosis Detection Kit and calculated as the percentage of PI/Annexin negative cells to total cells. Cardiac myocyte viability was analyzed on cells in gate R1 (Fig 6E), cardiac fibroblast viability was analyzed on cells in gate R2 (Fig 6F) and whole heart cells was analyzed with gate R1 plus gate R2 (Fig 6G). The quantitative data for cell viability were shown. Fig 6H showed that significant decrease of cardiac myocyte viability was found in I/R group compared with Sham (51.5 ± 2.4 versus 93.2 ±2.3, p<0.01), SalB-Rg1 considerately improved viability (61.5 ±2.4 versus 51.5 ± 2.4, p<0.05). No significant amelioration of SalB-Rg1 on cardiac fibroblast viability was found (Fig 6I). Considerable up-regulation of viability from whole heart cells was found with SalB-Rg1 treatment in comparison with I/R group, even though no statistic difference was detected (Fig 6J).

## Discussion

Reperfusion injury can offset the benefit of coronary blood flow restoration, especially in those with old age and prolonged ischemic times [13]. The present study unraveled that SalB-Rg1 combination showed significant cardio-protection against ischemia/reperfusion injury *in vitro* and *in vivo*. SalB-Rg1 not only improved heart function but also ameliorate heart structure, with the regulation on secretion of inflammatory cytokines and protection on cardiac myocytes during ischemia/reperfusion injury.

At present stage, no single known compound is potent enough to adequately protect the heart against ischemia/reperfusion injury, especially for patients with prolonged ischemic time. Combination for multiple compounds could impart more meaningful protection against infarction and achieve a greatly improved therapeutic outcome. SalB was reported to hold antioxidant, anti-arteriosclerotic, anti-inflammatory effects and to prevent angina pectoris and myocardial ischemia. Rg1 possesses the properties against myocardial remodeling, fatigue, apoptosis, and promotes angiogenesis for prevention and treatment of cardiovascular disease [14, 15]. In our study, SalB-Rg1 combination showed more significant effects on cardio-protection with histological and functional assessment than SalB or Rg1 treatment only, implying the therapeutic prospect for SalB-Rg1 combination to treat ischemia/reperfusion injury.

Myocardial reperfusion will lead to the opening of the mitochondrial permeability transition pore, result in the dissipation and uncoupling of the electrochemical gradient of the inner mitochondrial membrane, release highly destructive reactive oxygen species consequently [7, 16]. This oxidative stress leads to cardiac myocyte apoptosis which is a major contributor to ischemia/reperfusion injury [16]. The protective effect of SalB-Rg1 was first validated in H9c2 cells after establishment of the ratio of SalB to Rg1 as 2:5. SalB-Rg1 showed considerable efficiency against dissipation of the mitochondrial trans-membrane potential detected using JC-1, and further protected H9c2 cell from necrosis and apoptosis. Cell death is the prominent inducer for size of myocardial infarction. It is well accepted that infarct size correlates with arrhythmia severity, the development of heart failure and mortality. Improvements in outcomes have been made through facilitated reperfusion in conjunction with thrombolysis and percutaneous coronary intervention and the reduction of infarct size is a worthy goal in the treatment of myocardial infarction. SalB-Rg1 treatment not only protected cell death *in vitro*, but also considerably decreased infarct size of rat with ischemia/reperfusion injury *in vivo*, suggesting the promising prognosis.

Endothelial barrier dysfunction and subsequent intramyocardial haemorrhage are two well-recognized responses of the microvasculature dysfunction to cardiac I/R injury, results in an increased production of reactive oxygen species and soluble inflammatory mediators [17,18]. We observed the emigration of red cells from microvessels and up-regulation of several inflammatory cytokines in I/R rats. SalB-Rg1 not only considerably inhibited red cells leakage from microvessles but also down-regulated the expression level of several cytokines including TNF-α, IL-1β, RANTES, ICAM and sVCAM-1, suggesting the protection of SalB-Rg1 on endothelial barrier accompanying with their regulation on inflammatory cytokines.

The increased permeability appears to reflect the microvascular dysfunction that occur during both the ischemic and reperfusion phases. Chemokines including TNF-α, IL-1β, RANTES, ICAM and sVCAM-1, have been implicated in the increased vascular permeability induced by I/R injury [5,19,20]. The contribution of TNF-α to ischemia/reperfusion-induced microvascular dysfunction has been demonstrated using monolayers of cultured brain endothelial cells exposed to simulated flow cessation and reperfusion [20,21]. In the pulmonary microvasculature, immunoblockade of MIP-1, ICAM and sVCAM-1 blunts the I/R-induced permeability response, whereas RANTES and MCP-1 antibodies do not afford protection [21]. The down-regulation of SalB-Rg1 on cytokines such as TNF-α, IL-1β, RANTES, ICAM and sVCAM-1 was coordinated with its protection against microvascular permeability.

It is well-known that cardiac myocytes and cardiac fibroblasts are the two main cell types in the myocardium. Although, the proportion of each cell type varies among species and changes during maturation and in disease [22]. In general, cardiac myocytes make up no more than 50% and cardiac fibroblasts between 40 and 60% of the total cell population in the heart [23]. Reperfusion with arterial blood may salvage large numbers of damaged cardiac myocytes, but it has also been proposed that reperfusion itself can injure myocytes. The loss of cardiac myocytes is the major problem in heart failure; thus, it is important to protect cardiac myocytes against cell death during ischemia/reperfusion [24]. SalB-Rg1 increased viability of cardiac myocytes, reduced infarct size and improve functional parameters of the heart in rats, suggesting the cardio-protection of SalB-Rg1 with superior characteristics.

In summary, SalB-Rg1 exhibited significant improvement on cardiac function and structure in rats with ischemia/reperfusion injury. SalB-Rg1 is a potential medication with reasonable and effective indications for further clinical development.

## Supporting Information

S1 Fig Structure identification of SalB. (A) ^1^H NMR spectrum of SalB. (B) ^13^C NMR spectrum of SalB. (C) Chemical structure of SalB. (D) ^1^H and ^13^C NMR spectral data for SalB.
(TIF)

S2 Fig Structure identification of Rg1. (A) ^13^C NMR spectrum of Rg1. (B) Chemical structure of Rg1. (C) ^13^C NMR spectral data for Rg1.
(TIF)

S3 Fig The representative chromatogram of high-performance liquid chromatography. (A) SalB. (B) Rg1.
(TIF)

S4 Fig Experimental protocol for I/R injury in rats. (TIF)

## References

[1] Moran AE, Forouzanfar MH, Roth GA, Mensah GA, Ezzati M, Murray CJ, et al Temporal trends in ischemic heart disease mortality in 21 world regions, 1980 to 2010: the Global Burden of Disease 2010 study. Circulation. 2014;129:1483–1492. 10.1161/CIRCULATIONAHA.113.004042 24573352 PMC4181359

[2] Crea F, Battipaglia I, Andreotti F. Sex differences in mechanisms, presentation and management of ischaemic heart disease. Atherosclerosis. 2015;241:157–168. 10.1016/j.atherosclerosis.2015.04.802 25988360

[3] Poh KK, Xu X, Chan MY, Lee CH, Tay EL, Low AF, et al Safety of combination therapy with milrinone and esmolol for heart protection during percutaneous coronary intervention in acute myocardial infarction. Eur J Clin Pharmacol. 2014;70:527–530. 10.1007/s00228-014-1650-9 24463539

[4] Arslan F, Smeets MB, O'Neill LA, Keogh B, McGuirk P, Timmers L, et al Myocardial ischemia/reperfusion injury is mediated by leukocytic toll-like receptor-2 and reduced by systemic administration of a novel anti-toll-like receptor-2 antibody. Circulation. 2010;121:80–90. 10.1161/CIRCULATIONAHA.109.880187 20026776

[5] Guo J, Wang SB, Yuan TY, Wu YJ, Yan Y, Li L, et al Coptisine protects rat heart against myocardial ischemia/reperfusion injury by suppressing myocardial apoptosis and inflammation. Atherosclerosis. 2013;231:384–391. 10.1016/j.atherosclerosis.2013.10.003 24267256

[6] Feng GM, Chen JH, Lin CI, Yang JM. Effect of docosahexaenoic acid on hypoxia/reoxygenation injury in human coronary arterial smooth muscle cells. Eur J Nutr. 2012;51:987–995. 10.1007/s00394-011-0278-0 22105312

[7] Morrison A, Li J. PPAR-gamma and AMPK—advantageous targets for myocardial ischemia/reperfusion therapy. Biochem Pharmacol. 2011;82:195–200. 10.1016/j.bcp.2011.04.004 21536015

[8] Yue QX, Xie FB, Song XY, Wu WY, Jiang BH, Guan SH, et al Proteomic studies on protective effects of salvianolic acids, notoginsengnosides and combination of salvianolic acids and notoginsengnosides against cardiac ischemic-reperfusion injury. J Ethnopharmacol. 2012;141:659–667. 10.1016/j.jep.2011.08.044 21903157

[9] Wei YJ, Qi LW, Li P, Luo HW, Yi L, Sheng LH. Improved quality control method for Fufang Danshen preparations through simultaneous determination of phenolic acids, saponins and diterpenoid quinones by HPLC coupled with diode array and evaporative light scattering detectors. J Pharm Biomed Anal. 2007;45:775–784. 17720349 10.1016/j.jpba.2007.07.013

[10] Deng YP, Zhang TT, Teng FK, Li DF, Xu F, Cho K, et al Ginsenoside Rg1 and ginsenoside Rb1 play different roles in myocardial infarction rats in combination with salvianolic acid B. J Chin Med Assoc. 2015;78:114–120. 10.1016/j.jcma.2014.10.001 25476150

[11] Xu L, Deng Y, Feng L, Li D, Chen X, Ma C, et al Cardio-protection of salvianolic acid B through inhibition of apoptosis network. PloS one 2011;6:e24036 10.1371/journal.pone.0024036 21915278 PMC3167815

[12] Wang Y, Xu F, Chen J, Shen X, Deng Y, Xu L, et al Matrix metalloproteinase-9 induces cardiac fibroblast migration, collagen and cytokine secretion: inhibition by salvianolic acid B from Salvia miltiorrhiza. Phytomedicine. 2011;19:13–19. 10.1016/j.phymed.2011.06.024 21925853

[13] Betgem RP, de Waard GA, Nijveldt R, Beek AM, Escaned J, van Royen N. Intramyocardial haemorrhage after acute myocardial infarction. Nat Rev Cardiol. 2015;3:156–167. 10.1038/nrcardio.2014.188 25403783

[14] Xu H, Li Y, Che X, Tian H, Fan H, Liu K. Metabolism of salvianolic acid A and antioxidant activities of its methylated metabolites. Drug Metab Dispos. 2014;42:274–281. 10.1124/dmd.113.053694 24277725

[15] Zhou Q, Jiang L, Xu C, Luo D, Zeng C, Liu P, et al Ginsenoside Rg1 inhibits platelet activation and arterial thrombosis. Thromb Res. 2014;133:57–65. 10.1016/j.thromres.2013.10.032 24196231

[16] Diaz RJ, Fernandes K, Lytvyn Y, Hawrylyshyn K, Harvey K, Hossain T, et al Enhanced cell-volume regulation in cyclosporin A cardioprotection. Cardiovasc Res. 2013;98:411–419. 10.1093/cvr/cvt056 23483048

[17] Seif AA. Nigella sativa attenuates myocardial ischemic reperfusion injury in rats. J Physiol Biochem. 2013;69:937–944. 10.1007/s13105-013-0272-5 23846789

[18] Rodrigues SF, Granger DN. Role of blood cells in ischaemia-reperfusion induced endothelial barrier failure. Cardiovasc Res. 2010;87:291–299. 10.1093/cvr/cvq090 20299333 PMC2895540

[19] Wu B, Meng K, Ji Q, Cheng M, Yu K, Zhao X, et al Interleukin-37 ameliorates myocardial ischemia/reperfusion injury in mice. Clin Exp Immunol. 2014;176:438–451. 10.1111/cei.12284 24527881 PMC4008989

[20] Homma T, Kinugawa S, Takahashi M, Sobirin MA, Saito A, Fukushima A, et al Activation of invariant natural killer T cells by alpha-galactosylceramide ameliorates myocardial ischemia/reperfusion injury in mice. J Mol Cell Cardiol. 2013;62:179–188. 10.1016/j.yjmcc.2013.06.004 23774048

[21] Kassiri Z, Defamie V, Hariri M, Oudit GY, Anthwal S, Dawood F, et al Simultaneous transforming growth factor beta-tumor necrosis factor activation and cross-talk cause aberrant remodeling response and myocardial fibrosis in Timp3-deficient heart. J Biol Chem. 2009;284:29893–29904. 10.1074/jbc.M109.028449 19625257 PMC2785619

[22] Banerjee I, Fuseler JW, Price RL, Borg TK, Baudino TA. Determination of cell types and numbers during cardiac development in the neonatal and adult rat and mouse. Am J Physiol Heart Circ Physiol. 2007; 293: H1883–H1891. 17604329 10.1152/ajpheart.00514.2007

[23] Zhang P, Su J, Mende U. Cross talk between cardiac myocytes and fibroblasts: from multiscale investigative approaches to mechanisms and functional consequences. Am J Physiol Heart Circ Physiol. 2012;303:H1385–H1396. 10.1152/ajpheart.01167.2011 23064834 PMC3532535

[24] Suzuki YJ. Growth factor signaling for cardioprotection against oxidative stress-induced apoptosis. Antioxid Redox Signal. 2003;5:741–749. 14588147 10.1089/152308603770380043

