# Supplementary figures and images for: Combined Salvianolic Acid B and Ginsenoside Rg1 Exerts Cardioprotection against Ischemia/Reperfusion Injury in Rats
Source: PLoS One. 2015 Aug 17;10(8):e0135435. doi: 10.1371/journal.pone.0135435 (PMC4539231; doi:10.1371/journal.pone.0135435)

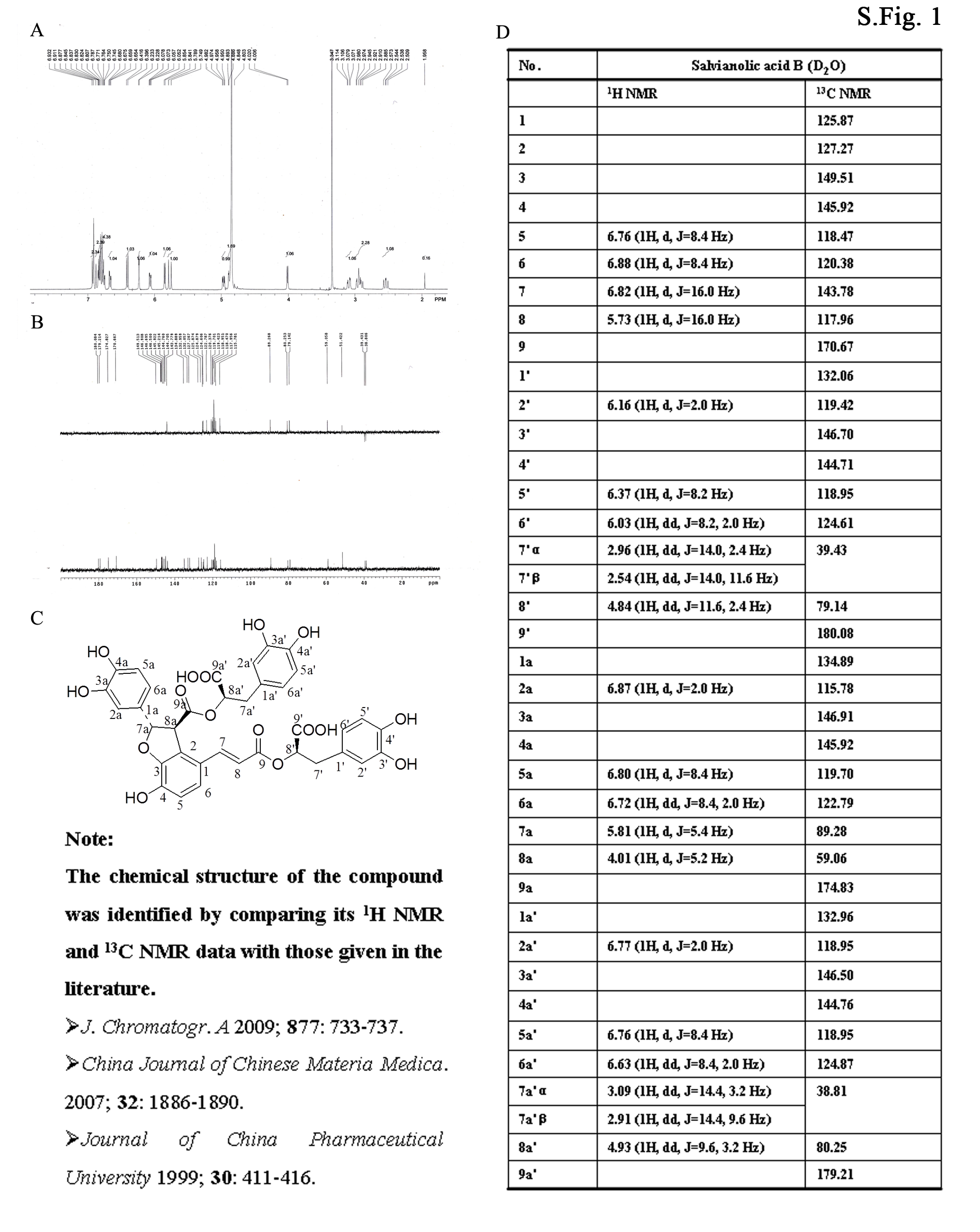

Supplement: S1 Fig — (A) 1H NMR spectrum of SalB. (B) 13C NMR spectrum of SalB. (C) Chemical structure of SalB. (D) 1H and 13C NMR spectral data for SalB. (TIF) [file pone.0135435.s001.tif]

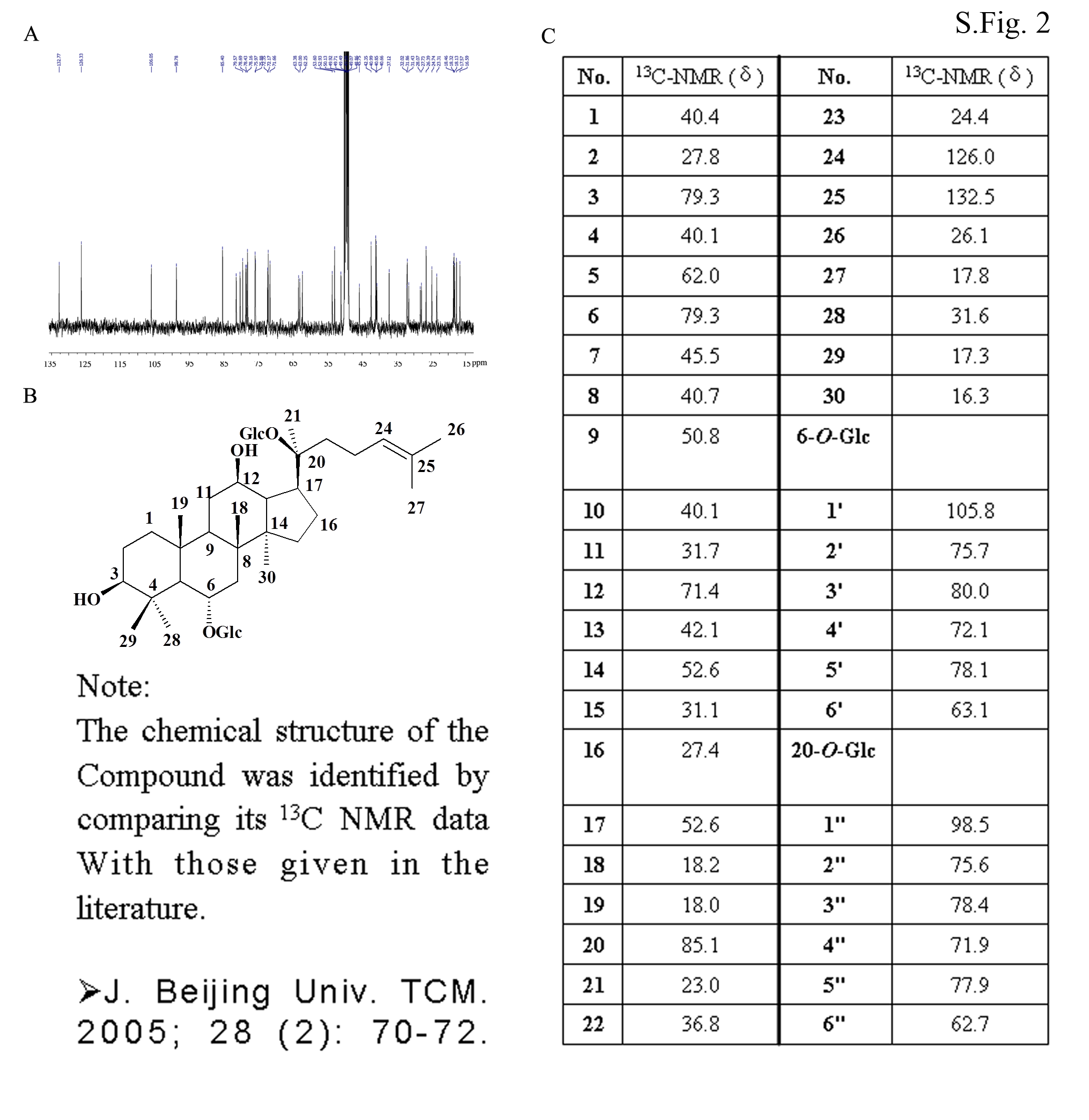

Supplement: S2 Fig — (A) 13C NMR spectrum of Rg1. (B) Chemical structure of Rg1. (C) 13C NMR spectral data for Rg1. (TIF) [file pone.0135435.s002.tif]

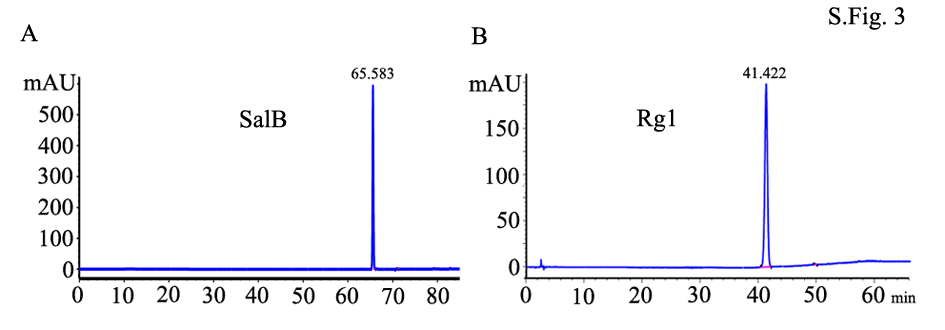

Supplement: S3 Fig — (A) SalB. (B) Rg1. (TIF) [file pone.0135435.s003.tif]

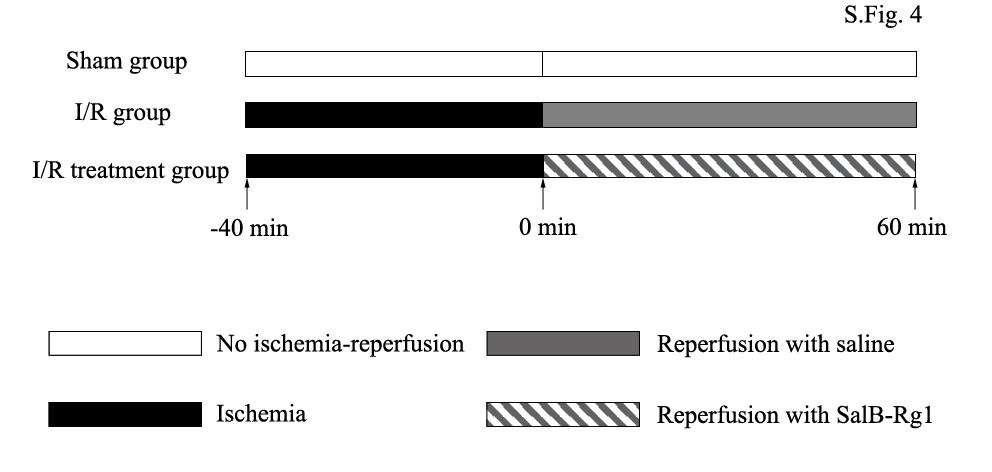

Supplement: S4 Fig — (TIF) [file pone.0135435.s004.tif]
